# Plaintes des patients: une opportunité pour améliorer la qualité des soins des personnes âgées avec des multimorbidités au Burkina Faso

**DOI:** 10.11604/pamj.2017.28.140.7297

**Published:** 2017-10-14

**Authors:** Hervé Hien, Macaire Ouédraogo, Abdramane Berthé, Blahima Konaté, Nadia Toé, Maxime koiné Drabo, Adiara Millogo, Fatoumata Badini-Kinda, Nicolas Meda, Jean Macq

**Affiliations:** 1 Centre MURAZ, Bobo-Dioulasso, Burkina Faso; 2 Institut de Recherche en Sciences de la Santé, Bobo-Dioulasso, Burkina Faso; 3 Centre Hospitalier Universitaire Sourô Sanou, Bobo-Dioulasso, Burkina Faso; 4 Laboratoire National de santé Publique, Ouagadougou, Burkina Faso; 5 Université de Ouagadougou UFR/SH, Burkina Faso; 6 Université Catholique de Louvain, IRSS, Bruxelles, Belgique

**Keywords:** Personnes âgées, plaintes, soins, formations sanitaires, pathologies chroniques, multi morbidités, Older people, complaints, care, health care facilities, chronic diseases, multimorbidities

## Abstract

**Introduction:**

Peu de données existent sur les plaintes des patients pour identifier des pistes d’amélioration de la qualité de soins des personnes âgées avec des multimorbidités. L’objectif de cette étude était d’analyser les plaintes des personnes âgées qui présentent des multimorbidités dans les formations sanitaires à Bobo-Dioulasso, Burkina Faso.

**Méthodes:**

Nous avons réalisé une étude transversale dans les formations sanitaires de la ville de Bobo-Dioulasso de novembre 2013 à février 2014. Les personnes âgées de 60 ans ou plus, avec au moins une pathologie chronique vues en ambulatoire ou en hospitalisation pendant la période d’étude ont été inclues. Des entretiens qualitatifs ont été réalisés à l’aide d’un questionnaire semi-structuré. Une analyse du contenu a été réalisée.

**Résultats:**

Nous avons noté des plaintes liées au long temps d’attente pour les soins, des conditions inadaptées de transfert des patients en hospitalisation, un manque d’échanges d’informations sur les maladies et des conditions d’hôtellerie inadaptées pour la personnes âgée venue en consultation et en hospitalisation.

**Conclusion:**

Des pistes d’amélioration pourraient passer par la rénovation et l’agrandissement des salles d’attentes des formations sanitaires, la séparation des prestations des soins chroniques des soins aigus en ambulatoire et en hospitalisation, l’appui à l’autonomisation par une meilleure communication avec le patient avec l’appui d’un groupe d’entraide communautaire et l’implication des familles.

## Abstract (en)

**Introduction:**

Little data exist on patient complaints to identify the strategy for the improvement of the quality of care of older people with multimorbidities. The aim of this study was to investigate the complaints of older people with multimorbidities at the health care facilities in Bobo-Dioulasso, Burkina Faso.

**Methods:**

We conducted a cross-sectional study in the health care facilities in Bobo-Dioulasso from November 2013 to February 2014. Older people aged 60 years or more, with at least a chronic disease, examined in ambulatory or in hospital during the study period were included. Qualitative interviews were conducted using a semi-structured questionnaire. A content analysis was performed.

**Results:**

We recorded the complaints related to long waiting time for health care, unsuitable hospital transfer service, lack of shared information on diseases and unsuitable hospitality conditions for older patients come to consultation and hospitalization.

**conclusion:**

Improvement strategies should include the renovation and extension of the waiting rooms in the health care facilities, the separation of chronic care practice from acute care practice in ambulatory and in hospital, the support to the empowerment through a better communication with the patient, a community mutual assistance group and the involvement of family members.

## Introduction

Actuellement, les pays du monde sont confrontés au phénomène du vieillissement de leur population [1]. Selon les estimations, les pays de l’Afrique subsaharienne sont très largement concernés par ce phénomène et devraient très vite adapter leur manière d’offrir les soins à ces populations. Ainsi, la qualité des soins des personnes âgées qui présentent plusieurs pathologies chroniques est un défi pour l’ensemble des systèmes de soins [2,3]. Ce nouveau paradigme des soins pour les personnes âgées qui présentent plusieurs pathologies chroniques, a suscité l’expérimentation de nombreux modèles de soins dans les pays développés dont l’un des plus connus est le modèle de soins chroniques [4]. Selon cette approche, le patient qui présente des multimorbidités, est considéré comme un partenaire. Il lui est offert des soins centrés sur ses besoins, un développement de son autonomisation et une l’implication de sa famille et de la communauté. Les multimorbidités sont définies comme la présence d’au moins deux pathologies chroniques chez la même personne que ce soit une coïncidence ou non [5,6]. Ces interventions qui visent à assurer la qualité des soins de longue durée font des progrès. Cependant, elles se heurtent encore à une faible identification des réelles priorités du patient [7]. Elles ne prennent pas suffisamment en compte l’avis des bénéficiaires pour la prise de décision et la planification opérationnelle des soins. La littérature suggère qu’une meilleure compréhension des priorités des soins par les personnes âgées elles-mêmes peut aider à la prise de décision et à la prise en charge appropriée et globale des multimorbidités [7,8]. En Afrique sub-saharienne et plus précisément au Burkina Faso, les tentatives des approches de soins des personnes avec des pathologies chroniques sont réalisées dans un contexte toujours centré sur la prise en charge des maladies et des problèmes aigus de santé. Par conséquent, elles pourraient être inadaptées sur le plan de l’accueil des patients, la communication interpersonnelle, la continuité de soins et l’environnement des soins. Ces insuffisances sont relevées par les systèmes de soins des pays développés qui ont expérimentés les soins modèles de soins des personnes âgées avec plusieurs pathologies [8-10]. Ces approches prenant en compte les besoins des patients n’ont pas encore été expérimentées dans notre contexte faute d’arguments scientifiques sur les besoins des personnes âgées. L’objectif de cette étude était d’analyser selon une approche qualitative les plaintes des personnes âgées qui présentent des multimorbidités dans les formations sanitaires à Bobo-Dioulasso, Burkina Faso.

## Méthodes

### Type et période de l’étude

Nous avons réalisé une étude transversale à prédominance qualitative de novembre 2013 à février 2014 dans les formations sanitaires de premier niveau et du CHU de la ville de Bobo-Dioulasso (Burkina Faso).

### Cadre de l’étude

Le système de soins du Burkina Faso comprend les sous-systèmes suivants: le sous-système public de santé, le sous-système privé et le sous-système de la médecine et de la pharmacopée traditionnelle. Les structures publiques de soins sont organisées en trois niveaux: le premier niveau est constitué par les centres de santé et de promotion sociale (CSPS), les centres médicaux (CM), les dispensaires et autres maternités isolées) et les hôpitaux de district de première référence. Le deuxième niveau comprend les centres hospitaliers régionaux (CHR) et enfin le troisième niveau regroupe les hôpitaux universitaires (CHU) [11]. L’étude a été réalisée dans la ville de Bobo-Dioulasso, deuxième ville du Burkina Faso. L’offre de soins au premier niveau est organisé par deux districts sanitaires. Ces 2 districts totalisaient 36 CSPS (27 pour le district sanitaire de Dafra et 9 pour le district sanitaire de Dô), 6 maternités ou dispensaires et 2 hôpitaux. Des structures de santé privées et confessionnelles participent à l’offre de soins. Le dernier niveau de référence est représenté par un CHU. Il n’y pas de niveau intermédiaire de soins à Bobo-Dioulasso [12].

### Population d’étude et critères de sélection des sujets

La population d’étude était constituée des personnes âgées fréquentant les formations sanitaires de l’étude. Ont été incluses dans l’étude, les personnes âgées de 60 ou plus, présentant au moins une pathologie chronique, vues en consultation ambulatoire, ou en hospitalisation et ayant donné leur consentement à participer à l’étude.

### Sélection des structures de santé

Nous avons sélectionné 6 structures de santé de manière raisonnée. Nous avons retenu les formations sanitaires sur des critères de fréquentation par les personnes âgées. Le choix des formations sanitaires a été guidé par les résultats d’une précédente enquête ménage sur les problèmes de santé des personnes âgées de la ville de Bobo-Dioulasso [13]. Les principales formations sanitaires dans lesquelles les personnes âgées avaient le plus recours ont été regroupées selon ce qui suit: le premier niveau de soins composé par deux Centres de Santé et de Promotion Sociale (CSPS), une clinique privé et deux hôpitaux de district de référence; Le niveau de référence le plus élevé de la ville: le service de la médecine interne du Centre hospitalier Universitaire (CHU).

### Sélection des personnes âgées

Les personnes âgées ont été sélectionnées de manière raisonnée. Nous avons interrogé consécutivement les personnes âgées de 60 ans ou plus qui se présentaient dans la formation sanitaire le jour de l’enquête soit qu’elles étaient venues en consultation ou référées d’une autre structure pour une hospitalisation. Pendant la période de l’étude, toutes les structures de santé ont été visitées chaque jour pour inclure les personnes âgées qui répondaient aux critères d’inclusion. Les prestataires ont été sollicités pour communiquer aux enquêteurs la présence d’une personne âgée venue en consultation ou en hospitalisation et répondant aux critères d’inclusion.

### Techniques et outils de collecte des données

*Des entretiens individuels* ont été réalisés avec les personnes âgées et /ou avec leur accompagnant si elles n’étaient pas à mesure de répondre aux questions. *Un guide d’entretien semi structuré* a été utilisé pour la collecte des données. Les items étaient: 1) les caractéristiques des personnes âgées, 2) l’accueil, 3) la communication avec le patient et son entourage, et 4) l’environnement de soins.

### Mode de collecte des données

Avec l’autorisation des responsables des formations sanitaires, les enquêteurs ont réalisé les entretiens avec les personnes âgées hospitalisées en dehors des heures de soins. Les entretiens avec les personnes âgées vues en ambulatoire se faisaient à la fin des consultations dans un local apprêté à ce effet. Elles étaient interrogées dans un endroit approprié (calme, dans une salle loin des regards des soignants). Quand une personne âgée venait en consultation ou était hospitalisée dans une formation sanitaire, le responsable du service de soins avertissait les enquêteurs par téléphone. La collecte des données a été réalisée par une équipe composée d’un médecin clinicien et d’une sociologue. Les entretiens ont été enregistrés avec la permission des enquêtés. Chaque entretien a duré environ une heure.

### Définition des concepts

*Pathologies chroniques et multimorbidités:* une pathologie chronique était définie comme ayant au moins une des caractéristiques suivantes: permanente, altération non réversible, nécessitant une réhabilitation, ou longue période de soins [14]. Les multimorbidités étaient définies comme la co-survenue de ≥ 2 maladies chroniques chez la même personne âgée [15].

*Accueil*: nous avons considéré l’accueil comme tout le dispositif ou les ressources matérielles, humaines, temporelles et émotionnelles disponibles dans la formation sanitaire pour le premier contact du patient avec la structure ou les prestataires.

*Communication interpersonnelle*: la communication interpersonnelle était définie comme le nombre et la qualité des interactions entre les prestataires et le patient ou son accompagnant pour son autonomisation (renforcement des connaissances du patient sur sa maladie et pour sa prendre en charge).

*Environnement de soins*: l’environnement de soins était constitué de l’hygiène des locaux, des points d’eaux, du système d’aération des salles, la disponibilité des sanitaires pour le confort des patients [16].

### Analyse des données

Tous les entretiens individuels ont été enregistrés à l’aide d’un dictaphone numérique, puis transcrits intégralement. Les entretiens transcrits ont été subdivisés en catégories élaborées a priori, puis encodées à l’aide du logiciel Nvivo 1.2. Le verbatim a été utilisé pour des illustrer les propos des participants. Une restitution des résultats a été faite avec les enquêtés et avec les chercheurs de la région qui s’intéressent à la thématique.

### Considérations éthiques et réglementaires

Le protocole de recherche a reçu l’accord du comité d’éthique pour la recherche en santé du Burkina Faso. Une notice d’information et un formulaire de consentement ont été utilisés pour recueillir le consentement des participants à l’étude. L’anonymat des personnes âgées a été préservé pendant les traitements des données. Des codes ont été attribués aux patients lors de la transcription des entretiens.

## Résultats

### Description de l’échantillon

Notre échantillon était constitué de 60 personnes âgées réparties en 46 vues en ambulatoire et 14 vues en hospitalisation. Dans le CHU, nous avons inclus 4 personnes âgées en hospitalisation. L’âge des enquêtés variait de 60 à 83 ans. Nous avons interrogé 23 femmes (Tableau 1).

**Tableau 1:** Caractéristiques des sujets enquêtés

Caractéristiques des personnes âgées (N=60)	n(%)
Age (moyen, SD) ans	68,1 +/- 5,9
**Sexe**	
*M*	**37 (61,7)**
*F*	23 (38,3)
**Statut matrimonial**	
*Mariés (vit en couple)*	**40 (66,7)**
*Célibataires*	2(3,3)
*Veuf (ves)*	18 (30)
**Pension de retraite**	
*Oui*	18 (30)
*Non*	**42(70)**
**Scolarisés**	
*Oui*	18 (30)
*Non*	**42 (70)**
**Provenance**	
*Urbain (Bobo)*	**51(85)**
*Rural*	9 (15)

### Les plaintes identifiées

### Accueil

L’accueil dans les formations sanitaires était mal apprécié par les personnes âgées. En effet, la quasi-totalité des personnes âgées ont déclaré s’être rendues très tôt pour leur âge, dans les formations sanitaires pour des soins. Aussi, l’attente était trop longue surtout dans les hôpitaux de districts pour leur âge. C’est dans ce sens que nous compris les propos de cette personne âgée de 72 ans de l’hôpital de district 1: « *je suis venu ici à 5h du matin et c'est vers 9 h que j'ai été reçu pour les soins* ».

Le long temps d’attente constitue également un risque de déséquilibre de certaines pathologies chroniques comme le diabète. Les propos de ce patient consultant au CHU l’illustrent « *Je suis diabétique, je viens à jeun à partir de 4h du matin et je ne vois le médecin qu’à 9h. Cela peut compliquer ma situation* » Pour cette personne âgée de 63 ans venue en consultation dans l’hôpital de district 2. « *L’attente aggrave la maladie. Il faut se lever à 4 h du matin pour avoir des soins, c’est difficile. Il faut trouver un autre endroit pour les personnes âgées ou privilégier les personnes âgées lors des soins*». Pour les patients hospitalisés, les conditions d’attente pour l’obtention d’une chambre d’hospitalisation étaient inadaptées comme le souligne ce patient de 65 ans dans l’hôpital de district 1. « *A notre arrivée j’ai dormi à même le sol en attendant que l’on me trouve une chambre avec un lit* ». Le transfert des patients vers les chambres d’hospitalisation était pénible et inadapté pour les patients déjà en incapacité fonctionnelle. C’est dans ce sens que nous avons compris les propos de ce patient de 76 ans en hospitalisation dans un hôpital de district 2: « *Mon fils m’a porté sur son dos pour m’amener en hospitalisation; parce que le brancardage était difficile; imaginez vous-même sous ce soleil avec toutes ces secousses; je n’allais pas tenir*».

### Communication lors des soins

Même si la plupart des personnes âgées interrogées ont reconnu un comportement de respect à leur égard, une bonne ambiance pendant la consultation et au cours de l’hospitalisation, des plaintes ont été notées sur la communication des informations sur leurs propres maladies, les conseils sur les maladies et les échanges avec leur accompagnant pour les aider à observer leur traitement. Plus de la moitié des patients interrogés ont déclaré n’avoir pas reçu des informations sur leurs pathologies. Les échanges étaient plutôt bien appréciés pour le personnel infirmier par rapport aux médecins. Les médecins communiquaient en français. Ainsi, la compréhension des messages était difficile. C’est dans ce sens que nous avons compris les propos de cette personne âgée de 67 dans le district sanitaire 1. « *Les médecins parlent en français. Comme je n’ai pas été à l’école, donc je ne comprends pas ce qu’ils se disent. Je n’ai pas compris* ». Les échanges se limitaient seulement à la prescription. Comme le souligne cette patiente de 74 ans dans un hôpital de district 1. « *Lors de la consultation, c’est d’abord l’ordonnance qu’il m’a donnée. Après il ne m’a plus rien dit. A chaque fois que je viens à sa consultation, il regarde les ordonnances, il ne me dit rien sur ma maladie* » Pour ce patient du CHU « *Je suis malade depuis 15 jours, je ne reçois que des médicaments, je ne suis pas soulagé et le pire je ne sais pas de quelle maladie je soufre*».

### Environnement des soins

La plupart des enquêtés ont déclaré que l’environnement de soins n’était pas approprié pour leur statut de personne âgée. Les toilettes de ces formations sanitaires étaient éloignées des locaux de la consultation. Dans les CSPS, les patients ont déclaré l’absence d’une salle d’attente pour les consultations. Quand elle existait, elle était restreinte, avec une insuffisance de places assises. C’est dans ce sens que nous avons compris les propos de ce patient de 71 ans consultant dans un hôpital de district 1. « *Il faut agrandir les salles d’attente, les personnes âgées ne peuvent pas attendre dans les mêmes conditions que les autres* ». Dans les hôpitaux de district, les motifs d’insatisfaction en hospitalisation étaient le nombre élevé des patients dans la salle, le non-respect de l’intimité, le manque d’hygiène, les toilettes éloignées des salles. Aussi, la mauvaise aération des salles rendait le séjour difficile comme le souligne cette patiente de 69 ans hospitalisée dans un hôpital de district 2. « *Cette salle sent très mauvais. Hier, nous avons demandé à changer de chambre pour nous seule même si nous devons payer plus. Mais les médecins nous ont dit qu’il n’y a pas de place. Alors que dans cette salle il y a beaucoup de gens* ».

## Discussion

### Principaux résultats

Au regard de nos résultats, nous avons noté des plaintes liées au long temps d’attente pour les soins, des conditions inadaptées de transfert des patients pour une hospitalisation, un manque d’échanges d’informations sur les maladies et des conditions d’hôtellerie inadaptées pour la personne âgée venue en consultation ou en hospitalisation.

### Les limites de l’étude

Les entretiens ont été réalisés au sein des formations sanitaires soit à la sortie de la consultation soit pendant l’hospitalisation. Cela peut laisser penser que les plaintes relevées par les patients pourraient être influencées par le cadre des entretiens. Nous avons utilisé des enquêteurs expérimentés, un cadre, un moment adéquat pour les entretiens individuels. Cela a pu créer un climat de confiance chez les enquêtés qui nous ont fourni des résultats intéressants sur leurs besoins de soins. Nous avons choisi d’aborder uniquement ces trois catégories qui nous semblaient importantes bien que d’autres soient également intéressantes à introduire dans notre modèle d’analyse pour explorer les plaintes de ce groupe cible.

### L’accueil

L’accueil est connu comme étant une faible caractéristique de l’organisation des soins dans nos systèmes de santé. Les salles d’attente dans les CSPS, les CMA ont une configuration standardisée. Elles ont été conçues pour répondre aux besoins des malades aigues qui nécessitent une prise en charge rapide et un séjour court dans la formation sanitaire. Cette insuffisance dans l’accueil des personnes âgées peut s’expliquer par la non séparation des patients âgés qui présentent des pathologies chroniques avec les autres qui présentent des pathologies aiguées. Ces deux typologies de pathologies ont des approches d’organisation de soins différente. Notre système d’offre de soins est toujours centré sur l’approche réactive qui oblige les prestataires à attendre les patients dans les formations sanitaires. Les soins sont administrés aux premiers venus sans tenir compte de leurs besoins et attentes géographiques (distance), physiques (incapacités fonctionnelles), cliniques (pathologies aiguées ou chroniques) et logistiques (transport vers le domicile ou dans la formation sanitaire). La répartition des tâches des prestataires obéit toujours à l’approche centrée sur la prise en charge des maladies. Les heures de consultation sont fixes pour chaque prestataire et pour un nombre de patients donnés. La conséquence est la précipitation des malades chez le prestataire qui assure la consultation du jour avec l’espoir d’être sur la liste des patients retenus quelque que soit sa pathologie ou son besoin. Les personnes âgées sont déjà fragilisées par le vieillissement de leurs organes et sont porteuses de plusieurs pathologies chroniques. Elles devraient bénéficier d’une autre approche d’organisation de soins. Malheureusement, elles sont toujours considérées au même titre que les autres malades qui viennent soit simplement pour des visites de surveillance pour les constantes ( poids, tension artérielle..), soit pour des pathologies qui nécessitent une prise en charge plus simple. Pour la prise en charge des personnes âgées avec des pathologies chroniques, le tri dès l’accueil pourrait être nécessaire pour les orienter vers des infrastructures et du personnel de soins adéquats [17]. Aussi, l’approche proactive par la planification anticipatoire des soins qui signifie que l’équipe de soins fixe expressément un rendez-vous de suivi médical avec son patient, sans attendre la survenue d’un épisode aigu [18] serait plus appropriée si elle est également accompagnée d’une stratégie de séparation des patients chroniques avec des aigues qui n’ont pas les mêmes besoins d’autonomisation.

### La communication interpersonnelle

La communication avec le malade chronique est une caractéristique du processus de soins qui pourrait contribuer à améliorer l’appui à l’autonomisation des personnes âgées. La participation du patient est importante et la prise en compte de ses besoins est nécessaire pour la planification des soins. Alexander, 2012, propose 4 principes dans l’approche de soins des personnes avec des pathologies chroniques dont la qualité des échanges est primordiale [19]. Selon lui, dans le cadre de la prise en charge des pathologies chroniques, la planification proactive repose sur les 4 principes suivants: 1) la qualité des échanges entre le patient et l’équipe de soins, 2) un traitement juste et respectueux du patient et centré sur ses besoins, 3) une participation du patient dans les objectifs du traitement, 4) la fréquence de la communication de l’équipe avec le patient à l’extérieure de la formation sanitaire. Dans notre système de soins, la communication se résume à la rédaction d’une ordonnance médicale pour le patient. Cela peut s’expliquer par le nombre élevé de patients qui attendent pour des soins devant la porte du prestataire, des rendez-vous des patients non planifiés, des multiples sollicitations du personnel médical pour des actes administratifs, des réunions non planifiées. Les conséquences sont la mauvaise la qualité de soins pour ce groupe spécifique qui a besoin de plus de temps pour l’écoute et les conseils pour les médicaments, les changements des habitudes de vie. Avec l’avènement des pathologies chroniques, la répartition des tâches et la planification des soins pourraient être repensées pour permettre au patient et au soignant de rentrer dans une démarche de communication plus longue et fructueuse.

### L’environnement des soins

La quasi-totalité des patients sont insatisfaits de l’environnement de soins. Cet inconfort est connu dans nos formations sanitaires et mal vécu par les personnes âgées qui présentent des multimorbidités [8]. Les pathologies chroniques nécessitent souvent de longs séjours d’hospitalisation dans les hôpitaux ou des ré-hospitalisation fréquentes pour la gestion des évènements intercurrents. La séparation des salles d’hospitalisation pour les personnes âgées accompagnée de l’hygiène des lieux devraient être des pistes pour améliorer l’hôtellerie de ce groupe cible. Cela permettait un désencombrement des salles et un meilleur séjour de ces personnes en respectant leur intimité. Pour l’hygiène des lieux c’est un problème qui constitue une source des risques infectieux chez les personnes qui sont déjà fragilisées. Hien, 2012 a réalisé une étude dans un hôpital de district du pays et avait déjà montré le niveau de risques infectieux que présentaient les malades hospitalisés. Il s’agissait de l’absence de point d’eau, de mauvaise qualité de l’air dans les salles, un mauvais système de ventilation des salles, une mauvaise hygiène des sols et des murs qui n’étaient pas régulièrement nettoyés [16]. Tous ces dysfonctionnements associés à la promiscuité dans les salles constituent des sources d’infections pour les personnes âgées avec des multimorbidités. Si l’appui à l’autonomisation des patients est bien développé dans les formations sanitaires, cela permettrait l’autogestion des soins à domicile (30), de retarder la fréquentation des formations sanitaires. Cela devrait permettrait une meilleure planification et une prise en charge de qualité pour ceux qui en ont vraiment besoin. Selon Sobel, si 5% des consultations médicales pouvaient être gérées à l’extérieur du service de santé, c’est-à-dire par les personnes elles-mêmes, la demande en services de santé seraient réduite de près de 25% [20].

## Conclusion

L’accueil infrastructurel (salle d’attente) et organisationnel (gestion des rendez-vous), la communication interpersonnelle pour l’appui à l’autonomisation, l’environnement décent pour l’hospitalisation au long cours sont les trois pièces d’un puzzle qui pourraient constituer des pistes pour l’amélioration du cadre de référence pour les soins de qualité des personnes âgées avec des multimorbidités. Ces pistes pourraient passer par la rénovation et l’agrandissement des salles d’attente des formations sanitaires, la séparation des prestations des soins chroniques avec les soins aigus en ambulatoire et en hospitalisation, l’appui à l’autonomisation par une meilleure communication avec le patient avec l’appui d’un groupe d’entraide communautaire et l’implication des familles et de la communauté. Ces pistes devraient apporter un bénéfice au-delà de ce groupe cible.

### Etat des connaissances actuelle sur le sujet

- Pour la prise en charge des personnes âgées avec des multimorbidités, le patient est un partenaire de soins;
- La prise en charge des patients avec des problèmes de santé chronique se fait selon une approche qui prend en compte les besoins des patients.

### Contribution de notre étude à la connaissance

- Il existe peu d’échanges entre les personnes âgées et les prestataires lors des soins;
- Les soins des personnes âgées se font selon une approche centrée sur la gestion des problèmes aigus;
- L’environnement des soins est inapproprié pour la prise en charge des personnes âgées déjà fragilisées par des multimorbidités.

## Conflits d’intérêts

Les auteurs ne déclarent aucun conflit d'intérêt.
